# Investigation on using high‐energy proton beam for total body irradiation (TBI)

**DOI:** 10.1120/jacmp.v17i5.6223

**Published:** 2016-09-08

**Authors:** Miao Zhang, Nan Qin, Xun Jia, Wei J. Zou, Atif Khan, Ning J. Yue

**Affiliations:** ^1^ Department of Radiation Oncology Robert Wood Johnson University Hospital, The Cancer Institution of New Jersey‐Rutgers University New Brunswick NJ; ^2^ Department of Radiation Oncology Division of Medical Physics and Engineering, University of Texas Southwestern Medical Center Dallas TX USA

**Keywords:** proton therapy, total body irradiation, Monte Carlo

## Abstract

This work investigated the possibility of using proton beam for total body irradiation (TBI). We hypothesized the broad‐slow‐rising entrance dose from a monoenergetic proton beam can deliver a uniform dose to patient with varied thickness. Comparing to photon‐based TBI, it would not require any patient‐specific compensator or beam spoiler. The hypothesis was first tested by simulating 250 MeV, 275 MeV, and 300 MeV protons irradiating a wedge‐shaped water phantom in a paired opposing arrangement using Monte Carlo (MC) method. To allow ±7.5% dose variation, the maximum water equivalent thickness (WET) of a treatable patient separation was 29 cm for 250 MeV proton, and >40 cm for 275 MeV and 300 MeV proton. The compared 6 MV photon can only treat patients with up to 15.5 cm water‐equivalent separation. In the second step, we simulated the dose deposition from the same beams on a patient's whole‐body CT scan. The maximum patient separation in WET was 23 cm. The calculated whole‐body dose variations were ±8.9%,±9.0%, ±9.6%, and ±14% for 250 MeV proton, 275 MeV proton, 300 MeV proton, and 6 MV photon. At last, we tested the current machine capability to deliver a monoenergetic proton beam with a large uniform field. Experiments were performed on a compact double scattering single‐gantry proton system. With its C‐shaped gantry design, the source‐to‐surface distance (SSD) reached 7 m. The measured dose deposition curve had 22 cm relatively flat entrance region. The full width half maximum field size was measured 105 cm. The current scatter filter had to be redesigned to produce a uniform intensity at such treatment distance. In conclusion, this work demonstrated the possibility of using proton beam for TBI. The current commercially available proton machines would soon be ready for such task.

PACS number(s): 87.53.Bn, 87.55.K‐, 87.55.‐x, 87.56.‐v

## I. INTRODUCTION

Total body irradiation (TBI) is commonly used as part of the bone marrow transplant preparative regimen for leukemia, aplastic anemia, or lymphoma treatment. The primary goal of TBI is to destroy the recipient's bone marrow and tumor cells, and to immunosuppress the patient sufficiently to avoid rejection of the donor bone marrow. In general, a uniform dose across the patient body is desired in TBI, though there are special situations in which lungs or other critical organs (e.g., kidney and bowel) should be partially shielded to lower the risk of radiation‐induced injury.^1^, ^2^

To achieve a uniform dose across patient's body with varied thickness, the ideal radiation beam should have a large field size, uniform intensity, as well as uniform dose deposition across varied depths. The current standard TBI treatment utilizes the linac‐based megavoltage photon beam. By placing the patient at extended source‐to‐surface distance (SSD) with maximum field opening, a large beam of relatively uniform fluence can be achieved from a modern linac. However, effort has to be made to ensure a uniform dose deposition across patient's body. As an uncharged particle, megavoltage photon's depth dose curve starts low, reaches the maximum after a short buildup, and then decreases. A common TBI setup uses two paired‐opposing beams with beam spoiler.^(3)^ The beam spoiler placed in front of the patient during treatment accommodates the dose buildup region to avoid underdosing patients' skin. However, since photon exponentially attenuates through the body, its dose distribution still varies with changing body thickness (e.g., higher dose to thinner part of the body, and vice versa). Patient‐specific compensators have to be designed to compensate for the body thickness variation. In practice, the compensator design is constantly challenged by large thickness variation, lack of immobilization, and internal tissue heterogeneities. Overall, ±10% dose variation can be achieved with careful planning and delivery.^(4)^

Proton has long been used for radiation therapy. A monoenergetic proton beam's depth dose curve has a broad‐slow‐rising entrance region before the sharp‐rising Bragg peak. The modern proton therapy takes advantage of the Bragg peak to treat the deeply seated tumor. The broad‐slow‐rising entrance, in other hand, is not used for any specific clinical application. However, we found the broad‐slow‐rising dose deposition is an idea beam for TBI: there is no dose buildup and the dose variation is small. If a monoenergetic proton beam with large uniform intensity is used to deliver TBI, as long as the maximum patient thickness is within the broad‐slow‐rising region, no beam spoiler or patient‐specific compensator would be needed to produce a uniform dose deposition in patient.

In this study, we tested our hypothesis of using proton beam for TBI. We evaluated different proton energies, patient thicknesses, and their relationships to dose variation. Aside from numerical investigations, we also did some pilot experiments on a proton therapy machine as a proof of concept.

## II. MATERIALS AND METHODS

### A. Water phantom study

Geant4 Monte Carlo toolkit^(5)^ was used in the simulation. Geant4 is a general purpose Monte Carlo code widely used in radiation therapy research. Its accuracy for proton dose calculation has been approved in numerous researches.^(6)^ A wedge‐shaped water phantom was designed in Geant4 to represent a patient with varied body thickness. The thickness varied from 2 cm to 40 cm linearly across 60 cm range.

Paired‐opposing beam arrangement with individual field size of 80cm×80cm were planned to deliver TBI on the simulated water phantom. Three proton beams with mean energies of 250 MeV, 275 MeV, and 300 MeV, plus 6 MV photon beam commonly used for linac‐based TBI, were tested. All the beams had uniform intensity across the field. The energy spectrum of each proton beam followed a Gaussian distribution with σ being 1% of the mean energy. The 6 MV photon beam had effective energy of 2 MeV. No beam divergence was simulated in any of those beams. A 1.5 cm thick water slab was placed in front of the phantom as a beam spoiler during the photon irradiation. Dose to the phantom was scored on voxels of $2\text{mm}\;\times 2\;\text{mm}\times 2\;\text{mm}.3.0\times 10^{8}$ source particles were transported for each proton beam, while $1.0\times 10^{9}$ source particles were transported for the photon beam to achieve less than 2% statistical uncertainty in dose.

In real clinical scenario, beam flatness and divergence affects the dose distribution significantly. However, since there is no real proton TBI beam available, the Monte Carlo simulation was performed using an ideal flat and nondivergent proton beam. This assumption was adequate for a theoretical investigation.

### B. Patient case simulation study

A set of whole‐body CT scans of one patient who received photon‐based TBI treatment in our center was used for the study. The maximum body thickness along the anterior–posterior direction was $23.0\;g/{\text{cm}}^{2}$ with the minimum of $5g/{\text{cm}}^{2}$. We calculated dose depositions on the CT scans from the same four beams as we tested in the water phantom study. The beam property, beam arrangement, and irradiation setup was the same as in the water phantom study. GPU‐based Monte Carlo codes for proton dose calculation^(7)^ and for photon dose calculation^8^, ^9^ were used. The computation resolution was 0.137cm×0.137cm×0.5cm matching the CT resolution. Dose to water was presented in the final results.

### C. Measurement

Mevion S250 proton therapy system (Mevion Medical System, Littleton, MA) is installed in our center. The system utilizes a superconducting magnet synchrocyclotron with passive double scattering method for beam delivery.^(10)^ The accelerator is mounted on a C‐shaped open gantry which can rotate from −5° to 185° following International Electrotechnical Commission (IEC) standards. The superconducting magnet synchrocyclotron produces a monoenergetic proton beam with the nominal energy of 250 MeV. The field shaping system of Mevion S250 generates 24 unique options of double‐scattered proton beam with different field sizes and penetration depths. Two different circular applicators are available with outer field diameters of 14 and 25 cm at the isocenter located at 200 cm nominal source‐to‐axis distance (SAD). Since the system has an open gantry, an extended treatment distance could be achieved by placing the subject at the wall with gantry at 90°. In our treatment room, the maximum possible treatment distance is 7 m. The Mevion S250 proton therapy system is currently not designed for TBI treatment (e.g., the double scatter filter was not designed to produce a uniform field at extended treatment distance). However, with open gantry design and double scattering beam delivery mechanism, it could potentially be modified to deliver a large uniform field with monoenergetic proton beam. In this study, we investigated the field size limitations and depth dose properties under current machine design. By doing this, we hoped to learn the current machine capability and the room for improvement.

**Figure 1:**
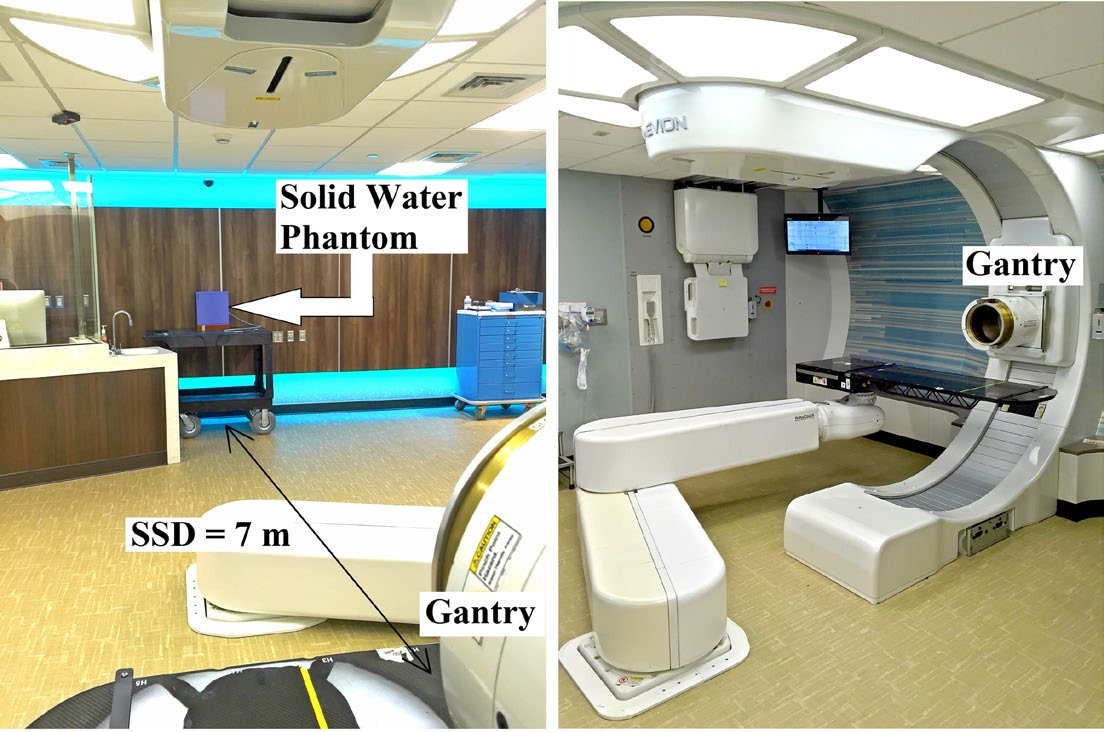
The extended treatment distance measurement on a Mevion S250 proton therapy system. With gantry angle at 90°, 7 meters of source‐to‐surface distance (SSD) can be achieved by placing the phantom against the back wall.

Among the 24 beam options, option 1 produces the largest field size of 25 cm diameter at the isocenter and the largest range of 25 cm in water. Due to its largest field size and range, option 1 was identified as the most suitable option for the possible TBI. Its beam properties at 7 m treatment distance were characterized by measuring the dose profile and the percentage depth dose (PDD) curve. A strip of Gafchromic EBT2 film (Ashland Inc., Wayne, NJ) was taped horizontally to measure the lateral dose profile. A solid water phantom with an ion chamber, as showing in Fig. 1, was used to measure the depth‐dose curve. The chamber was kept at 7 m from the source, while solid water slabs were added in front to change its depth in the phantom. To produce a monoenergetic proton beam, the range modulation wheel was removed from the beamline during the irradiation. No block was placed on the applicator to ensure the largest possible field size.

## III. RESULTS

### A. Water phantom simulation results

**Figure 2:**
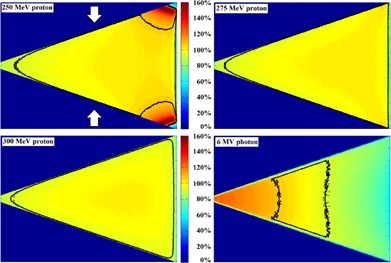
Central plane dose distributions from paired opposing 250 MeV proton, 275 MeV proton, 300 MeV proton, and 6 MV photon beams. The white arrows in the first graph represent the beam arrangement. The same color map was used for all the subplots. With dose normalized to the center of the phantom, the black contours identified the 100%±7.5% dose regions.

Figure 2 shows the dose distributions from all tested beams across the central plane of the wedge‐shaped water phantom. Each dose distribution was normalized to the center of the phantom. The thick black contour identifies the 100%±7.5% dose region. For 250 MeV proton beams, the very high dose regions at the thick corners of the water phantom were produced by the Bragg peak. The maximum phantom thickness allowed before the extreme high dose from the Bragg peak entering the phantom was $32\;g/{\text{cm}}^{2}$. When increasing the energy, protons traveled further before the Bragg peak appeared. For 275 MeV and 300 MeV protons, Bragg peaks were not observed within the water‐equivalent phantom when the separation exceeding $40\;g/{\text{cm}}^{2}$.

**Figure 3:**
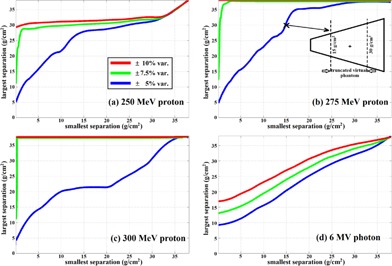
Isodose variation (IDV) lines for all tested beams. The IDV lines were used to characterize the largest phantom separation and the smallest phantom separation which allowed the beam to produce a dose distribution within a given dose variation criteria. The legend in (a) applies to all others subplots. The inset in (b) demonstrated how a virtual phantom for min/max separation of $15\;g/{\text{cm}}^{2}/30\;g/{\text{cm}}^{2}$ was generated.

To characterize the phantom thickness limits for a beam to deliver TBI, we plotted the isodose variation (IDV) curves in Fig. 3. The IDV curves were used to characterize combinations between a given beam and phantom thickness as the largest separation and the smallest separation, in which the beam can produce a dose distribution following a given criteria. For example, in Fig. 3(a), a point on the green line (±7.5% var.) of [10, 30] indicates for a phantom with the largest separation of $30\;g/{\text{cm}}^{2}$ along the beam direction and the smallest separation of $10\;g/{\text{cm}}^{2}$ or larger, the dose produced by a paired opposing 250 MeV proton beams would be between 92.5% and 107.5% if the dose was normalized to the center of the phantom. The IDV curves were generated from the simulated dose distribution of the wedge‐shaped water phantom. We sampled all the separation combinations within the range of $2\;g/{\text{cm}}^{2}$ to $40\;g/{\text{cm}}^{2}$ with $0.1\;g/{\text{cm}}^{2}$ resolution. In total we created 381×381=145161 virtual wedge phantoms. For a sampled virtual phantom (e.g., maximum separation of $30\;g/{\text{cm}}^{2}$ and minimum separation of $15\;g/{\text{cm}}^{2}$), the original phantom was truncated as the inset of Fig. 3(b) demonstrates. The dose to the truncated phantom was then derived from the original dose distribution with the same truncation with the assumption that the effect of proton lateral scattering at the edge of the phantom was negligible. The dose to the virtual phantom was then normalized to the center with the separation of $22.5\;g/{\text{cm}}^{2}$. Following this procedure, we calculated the total dose variation across all virtual phantoms. With all combinations in hand, we determined for a given total dose variation (e.g., 10%) and a given minimum separation, what would be the maximum phantom separation it allows to have and came up with the IDV curves. To truncate the original dose distribution to generate the virtual phantom dose distribution was an approximation. The dose at the edge of the phantom will be less in reality due to the lack of lateral scattering. However, the uncertainty introduced by such approximation would be acceptable considering high‐energy protons scatter less.

If we assumed the minimum patient separation in clinic was $5\;g/{\text{cm}}^{2}$ and we would accept ±7.5% dose variation, the IDV shows the maximum treatable patient separations were $29\;g/{\text{cm}}^{2}$, $>40\;g/{\text{cm}}^{2}$, and $>40\;g/{\text{cm}}^{2}$ for 250 MeV, 275 MeV, and 300 MeV proton beams, respectively. On the other hand, without tissue compensation, the maximum patient separation allowed for 6 MV photon beam was only $15.5\;g/{\text{cm}}^{2}$, which was far less than a typical patient separation.

### B. Patient phantom simulation results

**Figure 4:**
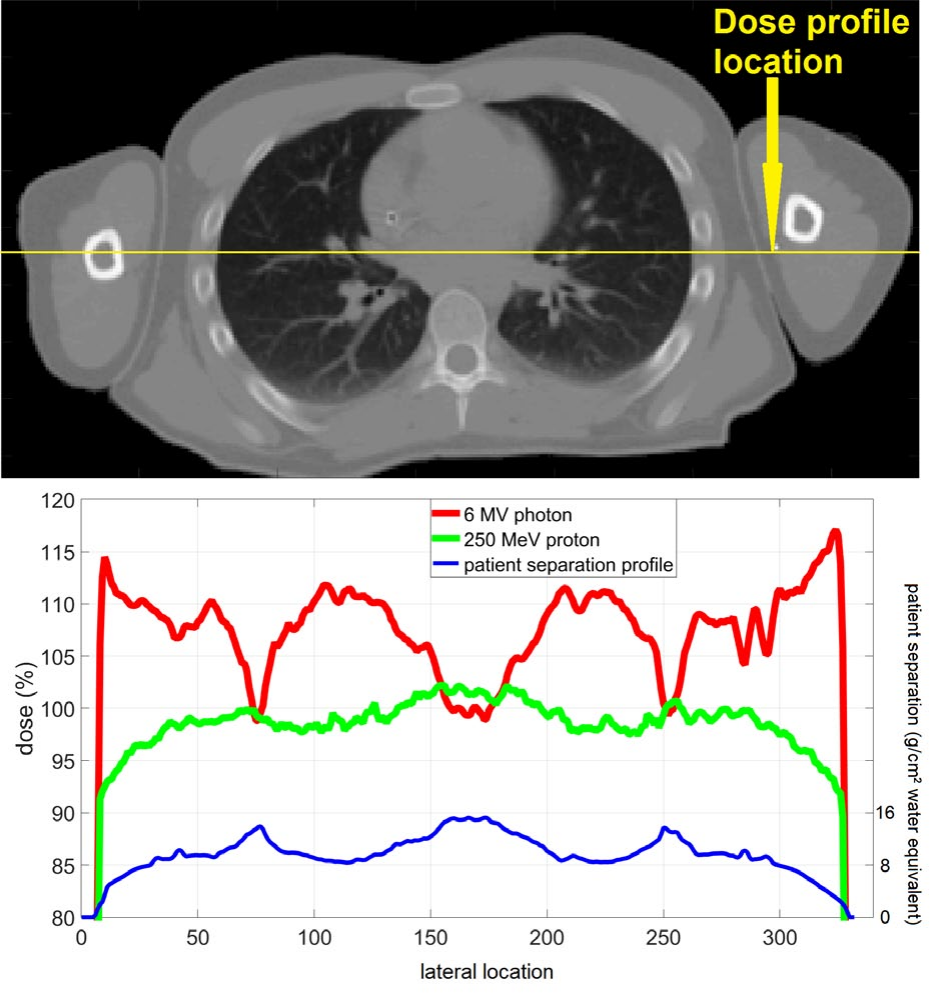
A CT slice showing mediastinum region (top) and the dose profiles of 250 MeV proton and 6MV photon (bottom). The location of the profile was marked in the CT image. The patient separation profile along the marked line was also plotted as a reference in the bottom graph.

Figure 4 shows lateral dose profile (dose to water) across one CT slide from a paired opposing 250 MeV proton beam and a 6 MV photon beam arranged along the anterior–posterior (AP) direction. The largest separation along the AP direction on this slice was $15.8\;g/{\text{cm}}^{2}$ located at the mediastinum. The separation reduced to $7.9\;g/{\text{cm}}^{2}$ by moving few centimeters laterally into the lung. The dose differences between those two locations was within 4% for the 250 MeV proton beam, whereas the 6 MV photon beam delivered 10% higher dose in lung than that in mediastinum.

**Figure 5:**
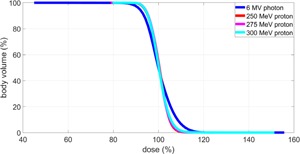
Whole‐body DVH of the TBI patient simulated with 250 MeV, 275 MeV, 300 MeV protons, and 6 MV photons. The DVH curves for all proton beams were heavily overlapping on the graph.

Figure 5 summarizes the whole‐body dose‐volume histogram (DVH) of all tested beams. The dose was normalized to the average dose of each case. The whole‐body dose variations calculated as ($D_{1\%}–D_{99\%}$)/2 were ±8.9%,±9.0%, ±9.6%, and ±14%, for 250 MeV proton, 275 MeV proton, 300 MeV proton, and 6 MV photon. Without any patient specific compensation, the protons' dose distributions were acceptable for TBI treatment (<±10%), whereas the photon's dose distribution was more heterogeneous.

### C. Measurement results

**Figure 6:**
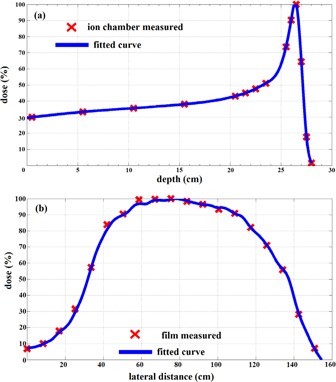
Ion chamber measured percentage depth‐dose curve (a) and film measured beam lateral profile (b) of beam option 1 without range modulation wheel from Mevion S250 proton system at 7 m treatment distance.

The entrance dose rate measured at the phantom surface was 0.95 Gy/min. The measured PDD at 7 m treatment distance is shown in Fig. 6(a). The curve was peaked at 26.4 cm depth in water, with the first 22 cm relatively flat and slow rising. Despite protons that traveled long distances in air, air scattering effect did not change the curve shape from a typical proton Bragg peak. The beam's lateral profile at 7 m treatment distance is plotted in Fig. 6(b). The curve was normalized to the maximum value. The full width half maximum (FWHM), >80% dose region diameter, and >90% dose region diameter were 105 cm, 77 cm, and 60 cm, respectively. Apparently, the beam profile was not flat and symmetric since the current machine was not designed to produce a flat treatment field at such a treatment distance. This result was only intended to demonstrate the current scatter design is capable to scatter 250 MeV protons into a large field with the help of a large treatment distance. However, for any real clinical application, the scatter needs to be tuned to produce a flat field. The design work is beyond the scope of current study and remains as a future research project.

## IV. DISCUSSION

There are various ways of delivering TBI nowadays: from the traditional compensator based static beam to modulated arc therapy^(11)^ or even Tomotherapy.^(12)^ The advanced techniques deliver more uniform dose distribution to patients for better treatment outcomes. However, dosimetry gains come with the increasing complexity during planning and delivery. With a broad‐slow‐rising entrance dose region, proton delivers uniform dose distribution without added complexity in planning or delivery. We have to point it out that the comparison between proton and photon in compensator free situation was not a fair game for photon since there are variety ways of modulating the photon beam intensity to deliver a uniform dose. To that extent, our intent was on showing the pure physics advantage of proton beams for TBI.

To design a proton system just to make TBI treatment easier would not be economically wise. However, with the increasing popularity of proton systems worldwide, there will be centers only equipped with proton modality. In that case, making proton therapy capable to treat a variety of diseases would be necessary. This study proved the concept of using the broad‐slow‐rising entrance dose from monoenergetic proton beam for TBI treatment. Comparing to photon‐based TBI, proton‐based TBI does not require patient‐specific compensator and beam spoiler to deliver uniform dose to the patient. Common factors causing dose variation (e.g., tissue inhomogeneity and patient motion) would be downplayed in this case, providing the maximum patient separation was carefully calculated considering all tissue heterogeneities as well as intrafractional motion caused separation change along the beam direction during planning. The whole planning process could be simplified to just determine the maximum patient separation and field size. However, between reality and the proposed application, there are several challenges that need to be addressed. First of all, proton energy is a limiting factor for the maximum treatable patient separation. For the mostly available commercial proton therapy systems, 250 MeV is the highest beam energy. Although a monoenergetic 250 MeV proton can treat up to $29\;g/{\text{cm}}^{2}$ water‐equivalent thickness, the maximum treatable separation would be reduced due to the presence of scatter filters in the beamline. For the tested proton system, the maximum treatable patient separation was about $22\;g/{\text{cm}}^{2}$. The typical patient separation along the anterior–posterior (AP) direction varies. In literature, one study^(13)^ mentioned the maximum AP separation of 23.3 cm among five patients. Meanwhile, the values from another study^(14)^ for 10 exceptionally large prostate patients were between 25 cm to 34 cm. Considering most TBI patients may weigh less than a large size prostate patient, 250 MeV double‐scattered proton beams would be adequate to treat the most of the TBI patients.

The second challenge for proton based TBI is the field size requirement. Scatter filters can be optimized to further increase the field size on any double‐scattering proton system. But increasing the treatment distance would be more efficient to achieve the same goal without sacrificing the proton energy for scatter filter. An open gantry system would have an advantage over the closed gantry system to achieve large treatment distances. Pencil beam scanning system using the steering magnetic may have the potential to produce a large field size without compromising the beam energy. However, its application may be limited by the dose rate requirement in TBI. Considering the radiobiological effects, TBI can only be safely conducted by delivering a whole‐body dose rate below 1 Gy/min.^(3)^ Compared to a double‐scattered proton beam system, a pencil beam from a pencil beam scanning system would deliver much higher dose rate to tissues its aiming at comparing to a double‐scattered proton beam. Although the overall whole‐body dose rate would be low due to the slow scanning through the whole body for pencil beam scanning, a high dose rate at each point may not be ideal radiobiologically.

The conceived proton‐based TBI requires the Bragg peak depositing beyond the patient which, without additional absorber, might reach the concrete wall of the treatment room and might produce harmful activated materials. To avoid this, a beam absorber made of low‐Z material (e.g., plastic) should be placed behind the patient to stop the proton beam.

## V. CONCLUSIONS

In this work, we propose the idea of using the broad‐slow‐rising entrance dose from monoenergetic proton beams to deliver TBI treatment. Monte Carlo simulation demonstrates the feasibility of delivering uniform dose distribution through a paired opposing beam arrangement. Comparing to conventional photon‐based TBI, proton‐based TBI would not require patient‐specific compensator to achieve a uniform dose distribution. Monoenergetic 250 MeV protons would be able to treat patients with body separation up to $29\;g/{\text{cm}}^{2}$. To achieve large treatment field sizes, the scattering filter needs to be modified in a double‐scattering proton system.

## ACKNOWLEDGMENTS

Authors would like to thank our onsite engineers, Mr. Dmitry Misyura and Mr. Troy Smith, for their help on delivering proton beams.

## COPYRIGHT

This work is licensed under a Creative Commons Attribution 3.0 Unported License.
